# The Prognostic Value of a Liver Function Test Using Indocyanine Green (ICG) Clearance in Patients with Multiple Organ Dysfunction Syndrome (MODS)

**DOI:** 10.3390/jcm13041039

**Published:** 2024-02-11

**Authors:** Franz Haertel, Sebastian Nuding, Diana Reisberg, Martin Peters, Karl Werdan, P. Christian Schulze, Henning Ebelt

**Affiliations:** 1 Department of Internal Medicine I, Cardiology, University Hospital Jena, Am Klinikum 1, 07747 Jena, Germany; 2 Department of Internal Medicine III, Cardiology University Hospital Halle (Saale), Ernst-Grube-Str. 40, 06120 Halle, Germany; 3 Department of Internal Medicine II, Cardiology, Hospital St. Elisabeth and St. Barbara Halle (Saale), Mauerstraße 5, 06110 Halle (Saale), Germany; 4 Department of pediatrics, Ameos Hospital Aschersleben, Eislebener Str. 7A, 06449 Aschersleben, Germany; 5 Department of Internal Medicine, Helios Hospital Jerichower Land, August-Bebel-Str. 55a, 39288 Burg, Germany; 6 Department of Internal Medicine II, Cardiology, Catholic Hospital “St. Johann Nepomuk”, Haarbergstr. 72, 99097 Erfurt, Germany

**Keywords:** acute liver failure, indocyanine green clearance, ICG, sepsis, intensive care, MODS

## Abstract

Background: Multiple organ dysfunction syndrome (MODS) is common in intensive care units (ICUs) and is associated with high mortality. Although there have been multiple investigations into a multitude of organ dysfunctions, little is known about the role of liver dysfunction. In addition, clinical and laboratory findings of liver dysfunction may occur with a significant delay. Therefore, the aim of this study was to investigate whether a liver function test, based on indocyanine green (ICG)-clearance, contains prognostic information for patients in the early phase of MODS. Methods: The data of this analysis were based on the MODIFY study, which included 70 critically ill patients of a tertiary medical ICU in the early phase of MODS (≤24 h after diagnosis by an APACHE II score ≥ 20 and a sinus rhythm ≥ 90 beats per minute, with the following subgroups: cardiogenic (cMODS) and septic MODS (sMODS)) over a period of 18 months. ICG clearance was characterized by plasma disappearance rate = PDR (%/min); it was measured non-invasively by using the LiMON system (PULSION Medical Systems, Feldkirchen, Germany). The PDR was determined on the day of study inclusion (baseline) and after 96 h. The primary endpoint of this analysis was 28-day mortality. Results: ICG clearance was measured in 44 patients of the MODIFY trial cohort, of which 9 patients had cMODS (20%) and 35 patients had sMODS (80%). Mean age: 59.7 ± 16.5 years; 31 patients were men; mean APACHE II score: 33.6 ± 6.3; 28-day mortality was 47.7%. Liver function was reduced in the total cohort as measured by a PDR of 13.4 ± 6.3%/min At baseline, there were no relevant differences between survivors and non-survivors regarding ICG clearance (PDR: 14.6 ± 6.1%/min vs. 12.1 ± 6.5%/min; *p* = 0.21). However, survivors showed better liver function than non-survivors after 96 h (PDR: 21.9 ± 6.3%/min vs. 9.2 ± 6.3%/min, *p* < 0.05). Consistent with these findings, survivors but not non-survivors show a significant improvement in the PDR (7.3 ± 6.3%/min vs. −2.9 ± 2.6%/min; *p* < 0.01) within 96 h. In accordance, receiver-operating characteristic curves (ROCs) at 96 h but not at baseline show a link between the PDR and 28-day mortality (PDR at 96 h: AUC: 0.87, 95% CI: 0.76–0.98; *p* < 0.01. Conclusions: In our study, we found that ICG clearance at baseline did not provide prognostic information in patients in the early stages of MODS despite being reduced in the total cohort. However, improvement of ICG clearance 96 h after ICU admission is associated with reduced 28-day mortality.

## 1. Introduction

Acute liver failure is a serious feature of multi-organ dysfunction syndrome (MODS) and continues to pose a significant diagnostic and therapeutic challenge. The sudden onset of liver impairment is commonly seen in critically ill patients, particularly those with sepsis, and the impact of liver dysfunction on clinical outcomes has been a topic of ongoing debate [1].

The liver, the largest gland in the human body, plays a crucial role in maintaining metabolic and immunological balance. It is responsible for over 200 functions, such as detoxification, storage, energy production, nutrient conversion, hormonal regulation, and coagulation. These functions make the liver a vital organ, and evidence has shown that liver dysfunction and failure, particularly in sepsis, can significantly contribute to disease progression and death [2].

**Figure 1:**
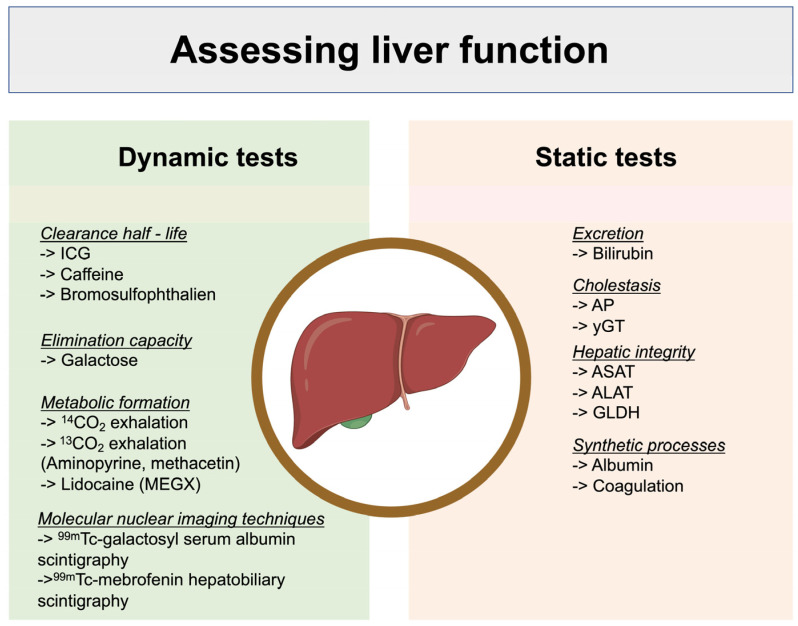
Comparison between dynamic and static liver function tests and a respective selection of test examples, modified according to Sakka et al. [15]. ICG = indocyanine green; AP = alkaline phosphatase, γ GT = gamma-glutamyltransferase; ASAT = aspartate transaminase; ALAT = alanine transaminase; GLDH = glutamate dehydrogenase (figure generated using BioRender©).

Assessing liver impairment can be challenging due to the many functions of this organ and the numerous parameters that can be measured. Static tests such as laboratory markers (ASAT = Aspartate transaminase; ALAT = Alanine transaminase etc.) can track chronic and acute impairments separately but cannot predict the quality or extent of functional recovery. Dynamic tests, on the other hand, provide functional information on the liver’s metabolism and clearance capacity (Figure 1) [3]. Indocyanine green (ICG) clearance is the most widely used dynamic test in clinical practice. ICG is a near-infrared fluorescent dye that is almost entirely eliminated by the liver and is not reabsorbed or metabolized [4]. It can be used to diagnose liver dysfunction, especially in pre- and post-operative patients undergoing hepatic surgery as well as in critical and non-critical patients in an ICU [1,5,6,7,8,9]. This method characterizes dynamic liver function as it depends on several factors such as liver blood flow, liver cell function, and biliary excretion [10,11,12,13,14].

### Aim of the Study

The main objective of this study is to investigate the relationship between ICG clearance, as an indicator of liver function, and the clinical outcomes of patients in the early stages of MODS.

## 2. Methods

### 2.1. Patients

The actual population was part of the prospective randomized controlled MODI_f_Y-trial population (EudraCT-Nr.: 2009-015499-88) that included critically ill patients with newly diagnosed MODS (inclusion ≤ 24 h of diagnosis by APACHE II—score ≥ 20) over a period of 18 months [15]. The study protocol of the MODI_f_Y trial was approved by the ethics committee of the Martin-Luther-University Halle (Saale), Germany [15]. The patients that were included had a study-independent indication for invasive hemodynamic monitoring, a sinus rhythm with a heart rate ≥ 90/min, pre-existing contraindications to beta blockers, and a signed declaration of informed consent. Exclusion criteria were as follows: age < 18 years, pregnancy or lactation, patients with chronic renal insufficiency (GFR < 30 mL/min), malignant hyperthermia, burns, acute rejection after organ transplantation, sick sinus syndrome, sinuatrial or atrioventricular block III°, cardiac pacemaker, high-grade valvular heart disease, severe hepatic failure, or suspected hypoxic brain damage after resuscitation. The included patients were prospectively stratified into cardiogenic (cMODS) and septic MODS (sMODS).

For our considerations, patients were examined twice over a period of 4 days. The ICU admission day corresponded to the day of the initial examination. A follow-up examination was carried out after 96 h. Pseudonymized demographic data, previous illnesses, vital and laboratory parameters, as well as clinical parameters were collected from all patients.

### 2.2. Study Endpoint

The primary clinical endpoint of this post hoc analysis was mortality within 28 days after ICU admission.

### 2.3. Conventional Parameters of Liver Function

Selected values of established parameters were study-independent routine clinical parameters collected post hoc outside of the MODI_f_Y study protocol and included the following: GGT = gamma-glutamyl-transferase (µmol/L·s); ASAT = aspartate transaminase (µmol/L·s); ALAT = alanine transaminase (µmol/L·s); INR = international normalized ratio; TB = total bilirubin (µmol/L).

### 2.4. Scoring Systems

To quantify the disease severity of the study population, we employed the Acute Physiology and Chronic Health Evaluation (APACHE) II score, which is designed to provide a morbidity score for ICU patients. Additionally, we utilized the Model of End-Stage Liver Disease (MELD) and MELD-NA scores to assess the severity of liver disease. 

### 2.5. Indocyanine Green (ICG) Clearance

**Figure 2:**
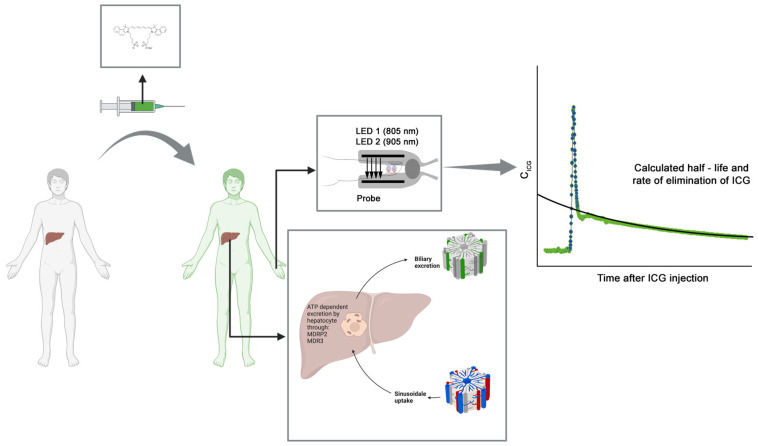
Study setup. Indocyanine green (ICG) is injected intravenously. After hepatic uptake, clearance takes place via biliary excretion through ATP-dependent pumps, involving multidrug resistance-associated protein 2 (MDRP 2) and multidrug resistance protein 3 (MDR3). Clearance is measured non-invasively using a probe with two light-emitting diodes (LEDs) with a wavelength of 805 nm and 905 nm. The relationship between the ICG concentration (C_ICG_) and the plasma disappearance rate (ICG-PDR) is described by the following equation: C_ICG_(t) = C_0_ × e^−PDR × t^ (figure generated using BioRender© and material from PULSION Medical Systems AG (Feldkirchen, Germany)).

ICG is a tricarbocyanine, fluorescent dye that has a high hepatic extraction rate of 70–80% (Figure 2) [16]. The liver extracts almost all of the ICG and transports it into the liver cells using transporting polypeptides (ATP-dependent export pump, multidrug resistance-associated protein 2 (MDRP2) and multidrug resistance P-glycoprotein (MDR3)); thus, ICG clearance reflects hepatic uptake/excretory function and energy status [17,18,19,20]. The ICG dilution curve after intravenous bolus administration shows a primary peak, which can be used to calculate cardiac output, a second elimination peak, also called the recirculation phase, which is sometimes followed by smaller peaks and is used to estimate circulating blood volume, and a hepatic elimination phase, lasting 10–20 min [5].

### 2.6. Global Liver Function Test via Non-Invasive Determination of ICG Clearance

The method used in this study to determine ICG clearance is based on the principle of pulse dye densitometry, which is similar to pulse oximetry [21]. Non-pulsatile and pulsatile readings are measured non-invasively through the skin, and the absorption ratios at corresponding wavelengths are calculated [21]. 

Pulse dye densitometry involves two light-emitting diodes (LEDs) [22]. One LED emits monochromatic light (red light), and the other LED emits light with a wavelength in the near-infrared range [22]. The absorption maximum for the dissolved ICG substance used in this study is approximately at a wavelength of 805 nm, which is also the isosbestic point of hemoglobin, where oxygenated and deoxygenated hemoglobin have the same extinction [22]. Therefore, the extinction of hemoglobin is neglected when measuring ICG clearance. The second LED serves as a reference in the near-infrared range, where ICG, unlike hemoglobin and other plasma components, has no absorbance. 

In this study, the ICG substance used was ICG-PULSION (PULSION Medical Systems AG, Feldkirchen, Germany), which is stored in a vial as a dark green powder. To preserve the ICG, it was kept under air conditioning at room temperature and protected from direct light exposure. A prerequisite for the measurement was venous access, such as a central venous catheter or venous cannula, which was checked for proper function before each measurement. 

The measurement of ICG clearance was performed at the patient’s bedside according to the manufacturer’s instructions. The technical setup involved a probe (LiMON, PULSION Medical Systems AG, Feldkirchen, Germany) and monitor (PiCCO2TM, PULSION Medical Systems AG, Feldkirchen, Germany). 

The ICG probe was placed on the patient’s finger via a clip and connected to the monitor. The next step was to administer an intravenous bolus (0.5 mg/kg body weight) of the ICG test substance dissolved in 10 mL of aqua ad injectabilia (Ampuwa^®^, Fresenius Kabi, Bad Homburg, Germany) followed by the administration of 20 mL of a 0.9% NaCl solution. After a few seconds, the curve of the ICG concentration (mg/L) versus time was displayed on the monitor, and results were obtained after approx. 20–25 min. 

### 2.7. Statistical Analysis

The data analysis and graph generation for this study was carried out using SPSS Statistics (version 26.0; SPSS Inc., IBM, Armonk, NY, USA) and GraphPad Prism 7.0 (GraphPad Software, La Jolla, CA, USA). The distribution of the data was analyzed using the Kolmogorov–Smirnov test and was presented as either the mean with standard deviation in case of normal distribution (mean ± SD) or the median with interquartile range in case of non-normal distribution (median (IQR, 25–75 percentiles)). For comparison of data with normal distribution, the Student’s *t*-test was used, while the non-normally distributed values were analyzed using the Mann–Whitney U-test. Differences between nominal scale variables were compared using the Chi^2^ test. The impact of ICG clearance on 28-day mortality was assessed using logistic regression and the respective odds ratios (ORs) including a 95% confidence interval (CI), both unadjusted and adjusted, as well as the area under the curve (AUC) values from the receiver operating characteristics (ROC) analysis. We derived changes in the PDR by computing the difference between the PDR measured after 96 h and the baseline PDR. A *p*-value of less than 0.05 was considered statistically significant.

## 3. Results

### 3.1. Patients

During a period of 18 months, 70 patients with MODS were enrolled in the MODIFY study at a tertiary medical 12-bed ICU. ICG clearance parameters were obtained in 44 patients. Table 1 lists the baseline characteristics of the study population according to the patient’s survival/ICG clearance status.

### 3.2. Demographic Data and Clinical Parameters at the Time of Hospital Admission

Data are given in Table 1. When comparing surviving and non-surviving patients, it is evident that neither demographic characteristics, such as age and gender, etc., nor clinical features, APACHE II score, MELD and MELD-Na scores, estimated glomerular filtration rate (eGFR), C-reactive protein (CRP), etc., show any significant differences. The only significant difference observed between the two groups were the albumin level (23.7 ± 7.5 g/L vs. 18.1 ± 6.9 g/L; *p* < 0.02) and the pH level (7.39 ± 0.1 vs. 7.27 ± 0.1; *p* < 0.01).

Table 1 also displays data regarding the study population categorized by their ICG clearance into normal (PDR > 18%/min) and impaired (PDR < 18%/min) subgroups, respectively. Both groups were similar in demographics including gender distribution and body mass index (BMI). Per definition, individuals with normal ICG clearance showed significantly higher PDR values (20.9 ± 3.1%/min) than those with impaired clearance (10.9 ± 4.9%/min, *p* < 0.01). MELD/MELD-Na scores, albumin levels, lactate, PCT, and γGT were in favor of the normal ICG clearance group. Additionally, those with normal ICG clearance displayed higher hemoglobin levels (7.5 ± 1.3 mmol/L vs. 6.3 ± 1.2 mmol/L, *p* < 0.02). However, there were no notable differences in inotropes and vasopressor doses or comorbidities.

### 3.3. Characterization of Liver Function 

**Figure 3:**
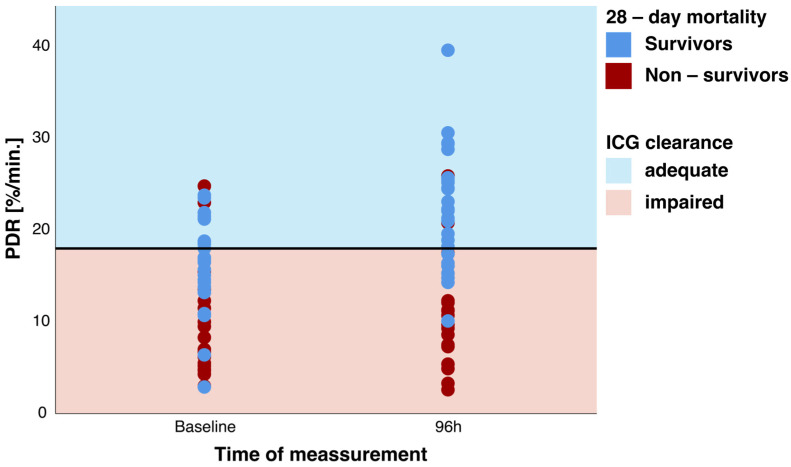
PDR at baseline and after 96 h in survivors and non-survivors with the respective classification of adequate and impaired indocyanine green (ICG) clearance. PDR = plasma disappearance rate.

Liver function as measured by ICG clearance was pathologically diminished in the studied population: mean PDR of 13.4 ± 6.3%/min at baseline. Although mean values are morbidly deranged, 11 patients (25%) could be identified to be in the adequate ICG clearance group (PDR > 18%/min) at baseline (Figure 3). Furthermore, 96 h after study inclusion, the number of patients with adequate ICG clearance increased to 22 patients (Figure 3).

### 3.4. Association between ICG Clearance and 28-Day Mortality

**Figure 4:**
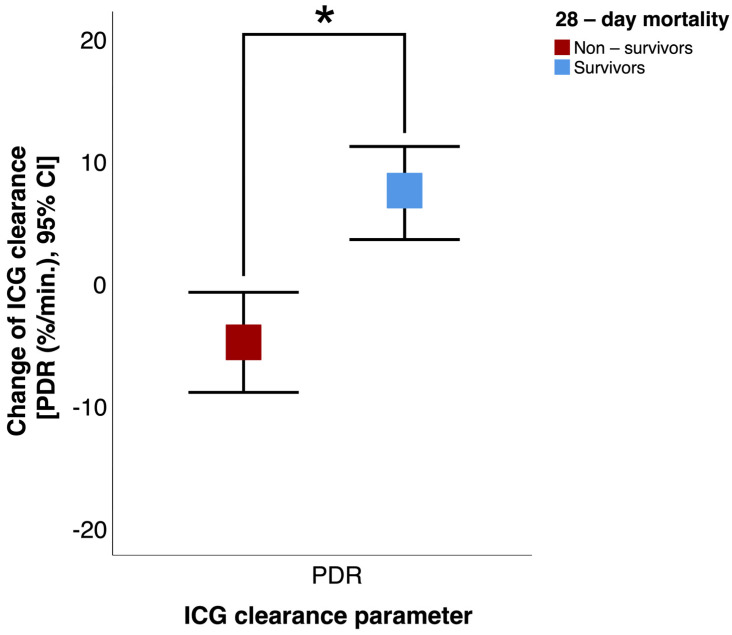
Change in indocyanine green (ICG) clearance (PDR, plasma disappearance rate) from baseline to 96 h in survivors and non-survivors. CI = confidence interval. * *p* < 0.05.

Significant differences between the survivors and non-survivors regarding their mean values could be found after 96 h: survivors had a higher PDR (21.9 ± 6.3%/min vs. 9.2 ± 6.3%/min; *p* < 0.05) than non-survivors. Survivors showed a significant improvement of ICG clearance within 96 h after baseline: PDR (+7.3 ± 6.3%/min vs. −2.9 ± 2.6%/min; *p* < 0.01) (Figure 4).

**Figure 5:**
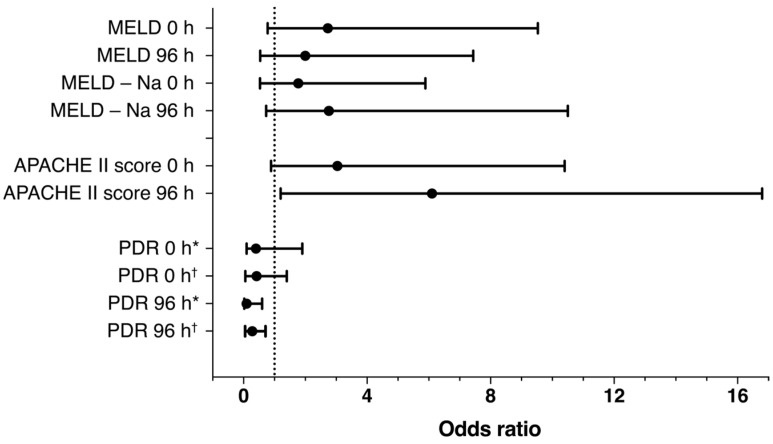
Forrest plot of binary logistic regression at baseline and after 96 h regarding the prediction of 28-day mortality. The following cut-offs were used for calculation: Acute Physiology and Chronic Health Evaluation (APACHE) II score: 33 points, Model of End Stage Liver Disease (MELD) score: 15 points; MELD-Na score: 17 points. Adjusted for APACHE II and MELD and MELD-Na scores at baseline or 96 h, respectively (* unadjusted; ^†^ adjusted).

Additionally, a binary logistic regression model was used to calculate the odds ratios regarding 28-day mortality; results are shown in Figure 5. For the PDR, a significant association can be obtained from a regression model after 96 h.

**Figure 6:**
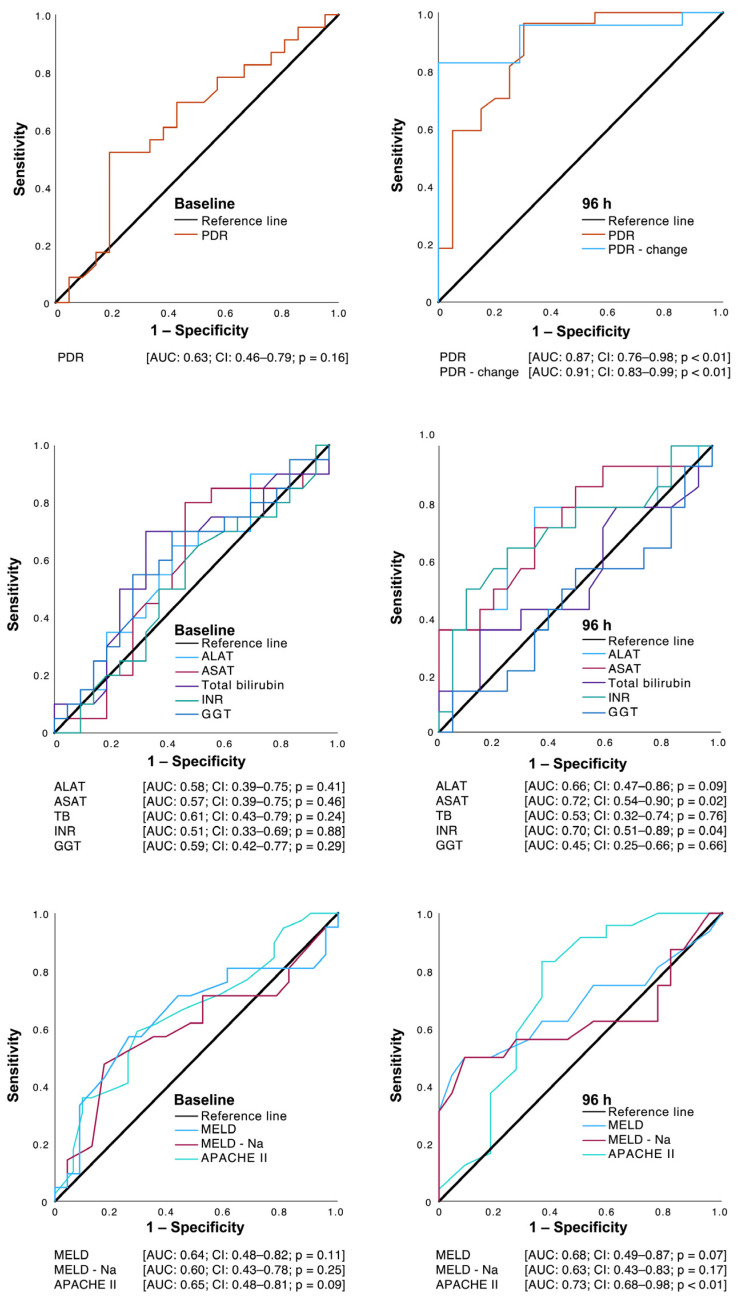
Receiver operating characteristic (ROC) curves of the indocyanine green PDR (plasma disappearance rate), as well as selected conventional parameters of liver function at baseline and after 96 h regarding the prediction of 28-day mortality. GGT = gamma-glutamyl-transferase; ASAT = aspartate transaminase; ALAT = alanine transaminase; INR = international normalized ratio; TB = total bilirubin; MELD = Model of End Stage Liver Disease; Na = sodium; APACHE II = Acute Physiology and Chronic Health Evaluation II.

Using ROC curves (Figure 6), relevant prognostic information regarding 28-day mortality can be found for the PDR and changes in the PDR as well as for the APACHE II score after 96 h. 

## 4. Discussion

The extent to which early changes in liver function in patients with new onset MODS can be detected and whether they have prognostic significance has not yet been studied sufficiently. The present study aimed to investigate whether the non-invasive determination of ICG clearance in patients with MODS is a suitable prognostic predictor for clinical endpoints. 

Sepsis-induced liver dysfunction plays a critical role in the progression of disease severity, as the liver is responsible for clearing infectious agents and products [23,24]. The main cause of the development of MODS in the present study population, with a proportion of 79%, is sepsis, contrasting past studies with heterogeneous populations encompassing surgical and medical patients [1,5,6,7,8,9]. 

The ICG clearance expressed as the PDR determined within 24 h after ICU admission is unsuitable for predicting 28-day mortality for MODS patients, according to our results. Findings from earlier studies suggest a relevant predictive potential either at study inclusion or ICU admission: Oellerich et al. [25] observed effective survival prediction in severe liver cirrhosis using ICG clearance at study inclusion. Similarly, Qui et al. [26] linked ICG clearance with post-hepatectomy liver failure (PHLF), correlating the test with PHLF occurrence and severity. Ishikawa et al. [27] demonstrated its predictive ability for post-operative complications in patients with hepatocellular carcinoma undergoing hepatectomy. However, a systematic review by Granieri et al. [28] noted variable sensitivity and specificity for ICG clearance in predicting PHLF, suggesting its limited reliability when used alone. Studies beyond liver-related diseases have also shown the predictive potential of ICG clearance. Weis et al. [29] found a strong correlation between pre-operative PDRs and mortality rates among cardiac surgery patients. Notably, investigations in septic patients have suggested ICG clearance as a potential prognostic marker. Inal et al. [6] identified the PDR as an effective predictor of mortality in septic patients in a surgical ICU. Similarly, Sakka et al. [30] and Kimura et al. [31] observed the PDR’s discriminative power in predicting ICU survival.

We were unable to replicate these findings, and this discrepancy could be partly explained by the notion that the initial insult during MODS may not have induced significant and measurable acute liver damage in our cohort. Other reasons could be attributed to the smaller patient number in our study. Also, our investigated population carries a relatively lower incidence of pre-existing liver dysfunctions/diseases than some of the aforementioned studies. 

Our approach to liver function testing included a follow-up ICG clearance measurement after 96 h, and the corresponding improvement was notably associated with the prediction of reduced mortality. We interpret this reversal of liver dysfunction as a consequence of the supportive treatment. Especially interventions aimed at sustaining adequate blood pressure (primarily through vasopressors), promoting effective cardiac output (mainly through inotropes, particularly dobutamine), and volume resuscitation help to address supporting liver perfusion/metabolism, while resolving the decline of a systemic, superior mesenteric artery and microcirculatory liver flow in the context of shock [32]. Our data suggest that liver function expressed through ICG clearance after 96 h has an independent effect on mortality as the corresponding odds ratios remain significant even after adjustment for the APACHE II and MELD/MELD-Na scores.

However, the clinical consequences of impaired liver function need to be carefully considered and judgment cannot rely on one parameter alone, such as the ICG clearance. Regarding a future aspect of ICG clearance, and though not performed in a MODS cohort, the PDR has been incorporated into an existing score (Model for End-Stage Liver Disease, MELD) in a previous study by Zipprich et al. with 604 patients [33]. The inclusion of ICG clearance into the MELD score (MELD-ICG) demonstrated improved discrimination in cases of intermediate to advanced cirrhosis (MELD score between 10 and 30) and allowed better prediction of survival in these patients than the MELD and MELD-Na scores alone, respectively. This is not surprising, as the MELD/MELD-Na scores themselves incorporate various serum parameters that do not represent real-time hepatic or kidney function. Serum creatine for example carries as a significant limitation a none-instantaneous reflecting of renal function: it can take 24–36 h to rise after a definite renal insult [34]. Furthermore, it has the tendency to overestimate renal function due to secretion in the proximal tubule [34]. Additionally, the administration of medications inhibiting tubular secretion can lead to an increase in creatinine levels, even in the absence of any actual change in renal function [34]. ICG clearance in this regard only represents hepatic blood flow and liver metabolism. ICG is entirely eliminated by the liver and is not reabsorbed or otherwise metabolized.

### Limitations of This Study

We acknowledge the inherent limitations of this retrospective analysis. Due to the design of the MODIFY study, all data were derived from patients with MODS so that no comparison with healthy controls can be given. Our study encompassed a diverse range of MODS patients, suggesting a multifactorial etiology for liver failure development. However, due to its small scale, this study lacks the statistical power necessary to comprehensively assess independent associations while adjusting for numerous factors. 

## 5. Conclusions

Early liver dysfunction associated with MODS is difficult to detect either by using conventional, static parameters or dynamic tests such as ICG clearance. Our data suggest that liver dysfunction manifests later during MODS, and then has a prognostic impact on the clinical outcome of these patients. The results presented provide evidence that a single measurement of ICG clearance at baseline carries no prognostic information in the early phase of MODS, but the PDR measurement after 96 h as well as the change of ICG clearance within 4 days of MODS are associated with 28-day mortality. At that time point, ICG clearance carries better prognostic information than standard liver blood tests or the MELD/MELD-Na score.

## Figures and Tables

**Table 1:** Baseline data of the total study population.

		28-Day Mortality			ICG Clearance		
	Total Study Population [*n* = 44]	Survivors [*n* = 23]	Non-Survivors [*n* = 21]	*p*-Value	Normal (PDR > 18%/min) [*n* = 11]	Impaired (PDR < 18%/min) [*n* = 33]	*p*-Value
**Demographics**							
Age [years, mean ± SD]	59.7 ± 16.5	58.4 ± 18.5	61.1 ± 14.3	n.s.	58.1 ± 16.5	60.2 ± 16.7	n.s.
<70 years [*n* (%)]	29 (66)	14 (61)	15 (71)	n.s.	8 (73)	21 (64)	n.s.
Male [*n* (%)]	31 (71)	17 (74)	13 (67)	n.s.	10 (91)	21 (64)	n.s.
BMI [kg/m^2^, mean ± SD]	26.1 ± 4.9	26.1 ± 4.8	26.9 ± 5.1	n.s.	26.7 ± 5.1	25.8 ± 4.9	n.s.
**Type of MODS at ICU admission**							
Cardiogenic MODS [*n* (%)]	9 (21)	6 (26)	3 (14)	n.s.	5 (45)	4 (12)	**<0.05**
Septic MODS [*n* (%)]	35 (79)	17 (74)	18 (86)		6 (55)	29 (88)	
**ICG clearance parameter**							
PDR [%/min, mean ± SD]	13.4 ± 6.3	14.6 ± 6.1	12.1 ± 6.5	n.s.	20.9 ± 3.1	10.9 ± 4.9	**<0.01**
**Clinical features and scores**							
APACHE II score [mean ± SD]	33.6 ± 6.3	31.9 ± 6.6	35.4 ± 5.5	n.s.	30.8 ± 4.9	34.5 ± 6.5	n.s.
MELD [mean ± SD]	18.9 ± 7.4	17.1 ± 6.9	20.5 ± 7.5	n.s.	12.9 ± 4.9	19.7 ± 7.1	**<0.01**
MELD-Na [mean ± SD]	18.5 ± 8.4	16.8 ± 7.7	19.7 ± 8.8	n.s.	12.3 ± 5.8	19.3 ± 8.6	**<0.02**
Creatinine level [µmol/L, mean ± SD]	178.3 ± 121.2	195.5 ± 139.3	149.7 ± 82.9	0.19	148.1 ± 107.6	182.2 ± 120.2	n.s.
eGFR [mL/min/1.73 m^2^, mean ± SD]	47.1 ± 32.6	41.3 ± 27,9	53.5 ± 36.7	0.22	56.6 ± 31.2	43.9 ± 32.9	n.s.
pH [mean ± SD]	7.30 ± 0.1	7.39 ± 0.1	7.27 ± 0.1	**<0.01**	7.35 ± 0.1	7.29 ± 0.9	n.s.
Body temperature [°C, mean ± SD]	36.8 ± 1.5	36.9 ± 1.5	36.7 ± 1.6	n.s.	36.8 ± 1.3	36.5 ± 1.5	n.s.
CRP [mg/L, mean ± SD	211.9 ± 161.1	183.3 ± 139.7	243.4 ± 179.7	n.s.	146.4 ± 98.9	233.8 ± 172.6	n.s.
Leucocytes [Gpt/L, mean ± SD]	16.9 ± 13.4	19.1 ± 13.9	14.3 ± 12.6	n.s.	12.3 ± 5.6	18.4 ± 14.8	n.s.
Albumin [g/L, mean ± SD]	21.1 ± 7.6	23.7 ± 7.5	18.1 ± 6.9	**<0.02**	29.4 ± 3.4	18.3 ± 6.6	**<0.01**
ASAT [µmol/L·s, median (IQR)]	1.4 (0.7–3.5	0.9 (0.6–3.9)	1.5 (1.0–3.4)	n.s.	0.75 (0.4–4.1)	1.5 (0.9–3.4)	n.s.
ALAT [µmol/L·s, median (IQR)]	0.7 (0.3–1.6)	0.5 (0.3–1.4)	0.9 (0.3–1.8)	n.s.	0.4 (0.2–2.1)	0.8 (0.3–1.6)	n.s.
INR [mean ± SD]	1.4 ± 0.7	1.4 ± 0.8	1.4 ± 0.5	n.s.	1.1 ± 0.1	1.5 ± 0.8	n.s.
GGT [µmol/L, median (IQR)]	1.2 (0.6–2.5)	0.9 (0.5–1.7)	1.7 (0.8–3.2)	n.s.	0.6 (0.3–2.2)	1.5 (0.9–3.5)	**<0.02**
Bilirubin [µmol/L, median (IQR)]	16.5 (11.3–26.3)	13.0 (11.0–21.0))	22.0 (11.5–37.1)	n.s.	15.1 ± 9.2	32.5 ± 41.5	n.s.
Lactate [mmol/L, median (IQR)]	1.8 (0.9–2.8)	1.7 (0.9–2.6)	1.9 (0.9–7.5)	n.s.	1.0 (0.8–1.7)	2.0 (1.1–5.4)	**<0.05**
PCT [µg/L, median (IQR)]	3.7 (1.1–8.1)	2.1 (0.4–4.9)	5.1 (1.4–9.3)	n.s.	1.4 (0.9–3.9)	4.8 (1.4–11.9)	**<0.05**
IL-6 [pg/mL, median (IQR)]	230 (83.4–1375.5)	180.2 (78.6–704.5)	1357.5 (97.0–2562.6)	n.s.	154.1 (74.5–705.3)	524.3 (76.2–2601.2)	n.s.
Invasive mechanical ventilation [*n* (%)]	40 (91)	19 (83)	21 (100)	n.s.	11 (100)	29 (87.9)	n.s.
Time of MODS diagnosis relative to ICU admission [hours, mean ± SD]	22.2 ± 19.1	19.9 ± 18.1	20.7 ± 20.4	n.s.	24.8 ± 18.2	21.3 ± 19.5	n.s.
Hemoglobin [mmol/L, mean ± SD]	6.6 ± 1.3	6.8 ± 1.3	6.4 ± 1.3	n.s.	7.5 ± 1.3	6.3 ± 1.2	**<0.02**
Norepinephrine dose [μg/kg/min, median (IQR)]	0.2 (0.03–0.6)	0.2 (0.07–0.6)	0.1 (0.07–0.4)	n.s.	0.1 (0.01–0.3)	0.4 (0.03–0.61)	n.s.
Epinephrine dose [μg/kg/min, median (IQR)]	0.018 (0.0–0.07)	0.01 (0.0–0.013)	0.03 (0.0–0.05)	n.s.	0	0.004 (0.0–0.14)	n.s.
Dobutamine dose [μg/kg/min, median (IQR)]	1.9 (0.0–4.04)	1.7 (0.01–3.33)	2.2 (0.0–4.86)	n.s.	1.4 (0.0–3.9)	2.6 (0.0–3.1)	n.s.
MAP [mmHg, mean ± SD]	77.3 ± 11.7	77.4 ± 12.1	77.2 ± 11.7	n.s.	73.7 ± 12.1	78.5 ± 11.5	n.s.
Heart rate [BPM, mean ± SD]	104.6 ± 16.8	104.2 ± 15.9	104.9 ± 18.1	n.s.	104.5 ± 16.1	104.6 ± 17.2	n.s.
LVEF [%, mean ± SD]	51.1 ± 12.8	53.8 ± 11.9	48.1 ± 13.4	n.s.	51.8 ± 11.8	49.5 ± 15.5	n.s.
**Comorbidities**							
Hypertension [*n* (%)]	20 (45)	10 (44)	10 (48)	n.s.	6 (55)	14 (42)	n.s.
Diabetes [*n* (%)]	13 (29)	8 (35)	5 (24)	n.s.	7 (64)	6 (18)	n.s.
CKD [*n* (%)]	5 (11)	3 (13)	2 (9)	n.s.	2 (18)	3 (10)	n.s.
Past myocardial infarction [*n* (%)]	11 (25)	6 (26)	5 (24)	n.s.	3 (27)	8 (24)	n.s.
Past stroke [*n* (%)]	1 (2)	1 (4)	21 (100)	n.s.	1 (9)	0	n.s.
COPD [*n* (%)]	8 (18)	2 (9)	6 (30)	n.s.	3 (27)	5 (16)	n.s.
AF [*n* (%)]	14 (32)	5 (23)	9 (45)	n.s.	4 (36)	10 (32)	n.s.
CAD [*n* (%)]	16 (36)	8 (35)	8 (38)	n.s.	6 (55)	10 (32)	n.s.
Past LTX [*n* (%)]	0	0	0	n.s.	0	0	n.s.
Chronic pancreatitis [*n* (%)]	1 (2)	1 (4)	0	n.s.	0	1 (3)	n.s.
Active alcohol abuse [*n* (%)]	8 (18)	5 (22)	3 (14)	n.s.	3 (27)	5 (16)	n.s.
Current Smoker [*n* (%)]	5 (11)	1 (4)	4 (19)	n.s.	2 (18)	3 (10)	n.s.
History of liver cirrhosis [*n* (%)]	6 (4)	3 (13)	3 (14)	n.s.	1 (9)	5 (16)	n.s.
Steatosis hepatis [*n* (%)]	6 (14)	3 (13)	3 (14)	n.s.	1 (9)	5 (16)	n.s.
Active malignancy [*n* (%)]	8 (18)	2 (9)	6 (29)	n.s.	1 (9)	7 (21)	n.s.

APACHE II-Score = Acute Physiology and Chronic Health Evaluation II-Score; BMI = body mass index; CRP = C-reactive protein; MODS = multiorgan dysfunction syndrome; CKD = chronic kidney disease; ICU = intensive care unit; PCT = procalcitonin; *n* = number of patients; n.s. = not significant; eGFR = estimated glomerular filtration rate; BPM = beats per minute; *p* < 0.05 = statistically significant, IQR = interquartile range, ICG = indocyanine green; LTX = liver transplantation; CAD = coronary artery disease; AF = atrial fibrillation; MAP = mean arterial pressure; COPD = chronic obstructive pulmonary disease; INR = international normalized ratio; ASAT = aspartate aminotransferase; ALAT = alanine aminotransferase; GGT = gamma-glutamyl-transferase; MELD = model for end-stage liver disease; Na = sodium.

## Data Availability

The data are not publicly available due to local legal restrictions on data safety.
